# The Ratio of Plasma and Urinary 8-oxo-Gsn Could Be a Novel Evaluation Index for Patients with Chronic Kidney Disease

**DOI:** 10.1155/2018/4237812

**Published:** 2018-01-17

**Authors:** Yong-Hui Mao, Leng-Nan Xu, Qing-Hua Weng, Xiang-Yu Li, Ban Zhao, Jing-Jing Nie, Ji-Hong Hu, Li-Qun Zhang, Zhe Chen, Ming-Zhang Zuo, Sadayoshi Ito, Jian-Ping Cai

**Affiliations:** ^1^Department of Nephrology, Beijing Hospital, National Center of Gerontology, Beijing 100730, China; ^2^The MOH Key Laboratory of Geriatrics, Beijing Hospital, National Center of Gerontology, Beijing 100730, China; ^3^School of Pharmacy, Wenzhou Medical University, Wenzhou, Zhejiang 325035, China; ^4^National Center for Clinical Laboratories, Beijing Hospital, National Center of Gerontology, Beijing 100730, China; ^5^Department of Anesthesiology, Beijing Hospital, National Center of Gerontology, Beijing 100730, China; ^6^The Second Department of Internal Medicine, Tohoku University School of Medicine, Sendai, Japan

## Abstract

Nucleic acid oxidation plays an important role in the pathophysiology progress of a variety of diseases. 8-oxo-7,8-dihydro-2′-deoxyguanosine (8-oxo-dGsn) and 8-oxo-7,8-dihydroguanosine (8-oxo-Gsn), which originate from DNA and RNA oxidation, were the most widely used indicators for oxidative stress. The study investigated the relation between 8-oxo-dGsn, 8-oxo-Gsn, and CKD. 146 patients with CKD were divided into five disease stages, and their fasting blood and morning urine were collected. The levels of 8-oxo-dGsn and 8-oxo-Gsn in plasma and urine were quantified by LC-MS/MS. The ratio of urinary 8-oxo-Gsn to creatinine increased from stages 1 to 4 corresponding to the increased severity of CKD, but it decreased in stage 5. And plasma 8-oxo-Gsn gradually increased with the decline of renal function. In particular, the increased ratio of plasma and urine 8-oxo-Gsn in stage 5 exceeded the concentration of creatinine. This trend was similar to the estimated glomerular filtration rate (eGFR), which indicates that 8-oxo-Gsn could be an appropriate indicator for renal function. Our finding indicates that as the disease progresses, RNA oxidation is increased. The significant increase in the ratio of plasma and urinary 8-oxo-Gsn is a novel evaluation index of end-stage renal disease.

## 1. Introduction

Reactive oxygen species (ROS) are persistently generated by living cells under normal physiological conditions as a consequence of cellular metabolism and external environmental factors, such as smoke and ultraviolet radiation. Previous studies have suggested that ROS can damage nucleic acids, lipids, and proteins. DNA and RNA precursor nucleotides are also subjected to oxidative damage. Among the various types of oxidized purine and pyrimidine bases thus produced, guanine has the lowest oxidation potential; it is most readily oxidized to form 8-oxo-7,8-dihydro-2′-deoxyguanosine (8-oxo-dGsn) and 8-oxo-7,8-dihydroguanosine (8-oxo-Gsn). The damaged nucleosides are produced in the cells, then circulated in the blood, and finally excreted in the urine. Oxidative damage of nucleic acids is a potential mechanism for the development of numerous diseases [1]. Rarely are oxidative products of nucleic acids studied in the field of renal disease, although they have been discussed in regard to diabetic nephropathy [2] or dialysis [3]. Patients in the predialysis group exhibited higher levels of nucleic acid oxidation, while oxidative levels were reduced after dialysis [3]. This revealed the important role of nucleic acid oxidation in renal disease. The study investigated the relation between 8-oxo-dGsn, 8-oxo-Gsn, and CKD.

CKD, defined by the presence of kidney damage (resulting in proteinuria) or reduced kidney function (estimated glomerular filtration rate (eGFR) < 60 mL/min/1.73 m^2^) [4], has become a serious, high-incidence disease that affects human health. According to epidemiology research, approximately 10 to 15% of the entire adult population worldwide have been diagnosed with CKD and it is estimated that CKD will increase to about 33% by 2020 [5–8]. Eventually, most patients will inevitably develop end-stage renal disease (ESRD). It is therefore urgent to find a promising diagnostic method for this disease.

In our research, we applied an accurate method based on isotope dilution ultra high-performance liquid chromatography triple-quadrupole mass spectrometry (UHPLC-MS/MS) to simultaneously assay the concentrations of 8-oxo-dGsn and 8-oxo-Gsn in the plasma and urine of 146 patients with CKD. Our data revealed the relevance of nucleic acid oxidative products in CKD diagnosis and could present a novel evaluation index for ESRD.

## 2. Materials and Methods

### 2.1. Chemicals

The 8-oxo-Gsn (>98% purity), 8-oxo-dGsn (>98% purity), and heavy-isotope-labeled 8-oxo-dGsn were obtained from Cambridge Isotope Laboratories (USA). The 8-oxo-[^15^N_2_^13^C_1_]Gsn was purchased from Toronto Research Chemicals (Canada), HPLC-grade ammonium acetate was purchased from Fisher Scientific (USA), and HPLC-grade solvents were obtained from Merck (Germany). Water used for the analysis was of Milli-Q quality (18.2 M*Ω* cm).

### 2.2. Patient Inclusion and Sample Collection

All subjects were enrolled in the department of Nephrology of Beijing Hospital, and all participants were adults (age ≥ 18 years) with a confirmed diagnosis of CKD. The 146 patients with CKD were divided into five disease stages according to the national kidney foundation guidelines, with approximately 30 participants in each stage. The diseases included in this study encompassed a variety of kidney diseases, except for diabetic nephropathy. Thus, the diseases included in the study were primary renal disease (IgA nephropathy, membrane nephropathy, minimal change disease, etc.); secondary renal disease (hypertensive nephropathy, gout nephropathy, lupus nephritis, etc.); renal tubular interstitial disease; and genetic diseases. All patients fasted before sample collection. Whole blood samples were collected in vacuum tubes pretreated with the anticoagulant Na_2_ EDTA and immediately centrifuged at 2500 rpm for 10 min. Plasma specimens (1 mL) were then isolated. Fresh urine specimens were obtained in the morning from midstream urine. Plasma and urine samples were frozen at −80°C before analysis.

### 2.3. Preparation of Plasma Samples

The frozen plasma specimens were thawed on ice. After vortexing for approximately 1 min, samples were centrifuged at 14000*g* for 10 min at 4°C. A 300 *μ*L aliquot of the supernatant was added to 10 *μ*L of 8-oxo-[^15^N_5_]dGsn and 10 *μ*L of 8-oxo-[^15^N_2_^13^C_1_]Gsn as an internal standard (300 pg/*μ*L); then after a brief mixing, a threefold volume of acetonitrile was added. Samples were vortexed for approximately 1 min, and then centrifuged at 14000*g* for 20 min at 4°C. The upper organic layer was quantitatively transferred (1 mL) into a tube and evaporated to dryness under a gentle steam of nitrogen. The dried residue was dissolved in 100 *μ*L of water and centrifuged at 12000*g* for 20 min at 4°C, and an 85 *μ*L aliquot was injected into the UHPLC-MS/MS system for analysis.

### 2.4. Preparation of Urine Samples

The frozen urine was thawed and heated at 37°C for 5 min and then centrifuged at 7500*g* for 5 min at 4°C. To each 200 *μ*L aliquot of the supernatant, 200 *μ*L working solution (70% methanol, 30% water with 0.1% formic acid, and 5 mmol/L ammonium acetate), 10 *μ*L 8-oxo-[^15^N_5_]dGsn, and 10 *μ*L 8-oxo-[15N213C1]Gsn as an internal standard (480 pg/*μ*L) were added. The mixture was incubated at 37°C for 10 min to redissolve the analytes from precipitate and was then centrifuged at 12,000*g* and 4°C for 15 min. Finally, 5 *μ*L of the supernatant was injected for UHPLC-MS/MS analysis. Because of the variability among the urinary volumes and the significant differences in the renal glomerular function, the concentrations of analytes were normalized relative to the amount of creatinine. An automatic biochemical analyzer 7600 series was used for determining the concentrations of urinary creatinine.

### 2.5. Chromatographic and Mass Spectrometric Analyses

All samples were analyzed by an Agilent 1290 Infinity UHPLC instrument equipped with an Agilent triple-quadrupole mass spectrometer with a Jet Stream ESI source and iFunnel (Agilent, 6490, USA). UHPLC separation was achieved on an Agilent C18 (3 *μ*m, 3.00 × 100 mm) column at 35°C using mobile phase A (5 mM ammonium acetate with 0.1% formic acid) and mobile phase B (methanol with 0.1% formic acid). The flow rate was 0.4 mL/min. The UHPLC conditions are shown in supplementary Table 1. To reduce interference and ensure the quality of the test results, we discarded early and late eluting components. In addition, the sample room temperature was kept at 4°C to decrease the loss of sample solution.

Mass spectrometric data acquisition was in the positive ion detection mode. Optimum nitrogen pressure for the nebulizer was 30 psi and ESI needle voltage was adjusted to 2000 V. The temperatures of dry gas and sheath gas were set at 200°C and 400°C, respectively, and the flow rates of dry gas and sheath gas were16 L/min and 12 L/min, respectively. Multiple reaction mode (MRM) was monitored for quantitative analysis. The high-pressure RF and low-pressure RF were 120 V and 50 V, respectively. UHPLC conditions and optimized parameters are presented in supplementary Tables 1, 2, and 3.

### 2.6. Statistical Analysis

Data are presented as the ratio of the concentrations of nucleosides (8-oxo-dGsn and 8-oxo-Gsn) and creatinine in urine. The continuous parameters are expressed as mean ± standard derivation (SD) and analyzed by SPSS Version 22.0. The values of *P* < 0.05 were considered statistically significant for all analyses. The results were assessed using one-way variance analysis, Spearman's correlation analysis, paired *t*-test analysis, and multiple regression analyses.

## 3. Results

In this study, 47.95% of the patients were male (*n* = 70). The age of the patients ranged from 21 to 88 years, and the average age was 56.43 ± 16.79 years. There were no statistical differences in gender distribution. Patients with stage 1 and 2 CKD were younger than those in the other three groups (*P* < 0.05). Considering the unique pathophysiological mechanism of diabetic nephropathy, we excluded those patients from the study. The greatest number of patients was suffering from primary renal disease, with secondary renal disease being the second most prevalent. The disease spectrum was similar in each stage (Table 1).

### 3.1. Creatinine (Cr), 8-oxo-dGsn, and 8-oxo-Gsn in Plasma and Urine

The levels of creatinine (Cr), 8-oxo-dGsn and 8-oxo-Gsn in fasting plasma and morning urine were measured in all stable patients to measure the level of nucleic acid oxidation. The results were showed in Table 2. The concentration of plasma 8-oxo-Gsn gradually increased and urine 8-oxo-Gsn decreased significantly (*P* < 0.05) with the decline of renal function. The ratio of urinary 8-oxo-Gsn to Cr (8-oxo-Gsn/Cr) increased from stages 1 to 4, which correlated with the severity of the disease. However, urinary 8-oxo-Gsn/Cr levels unexpectedly decreased in stage 5. There were significant differences between stages 1 and 4 in urinary levels of 8-oxo-Gsn/Cr (*P* = 0.044). Therefore, the ratio of 8-oxo-Gsn in plasma to urine increased, especially in stage 5.

### 3.2. 8-oxo-dGsn and 8-oxo-Gsn Had a Positive Correlation with Creatinine

**Figure 1:**
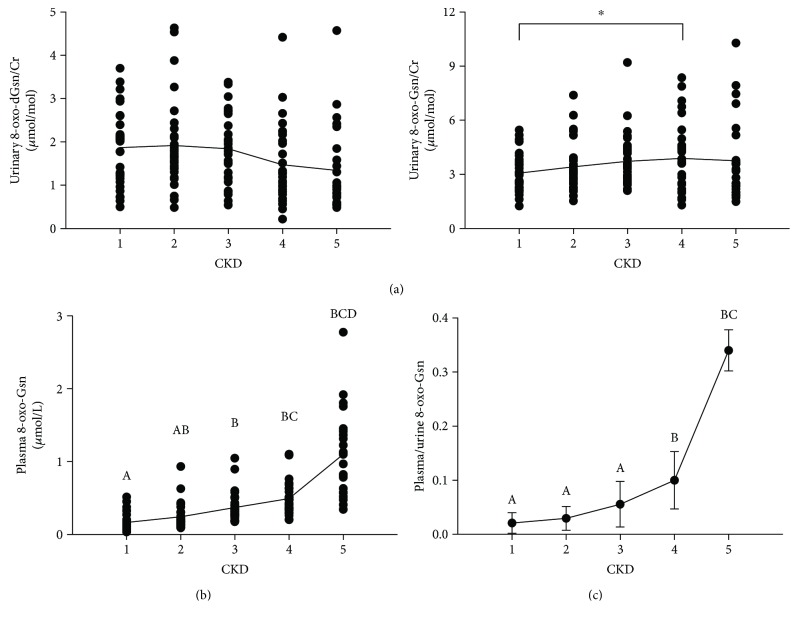
(a) Urinary 8-oxo-dGsn/Cr and 8-oxo-Gsn/Cr levels in CKD patients. There were significant differences in the levels of 8-oxo-Gsn between CKD1 and CKD4 (^∗^ *P* = 0.044). (b) Plasma 8-oxo-Gsn levels in CKD patients. Letters indicate statistical significance (*P* < 0.05). (c) The ratio of plasma and urinary 8-oxo-Gsn in patients with CKD. Letters indicate statistical significance (*P* < 0.05). CKD: chronic kidney disease; 8-oxo-dGsn: 8-oxo-7,8-dihydro-2′-deoxyguanosine; 8-oxo-Gsn: 8-oxo-7,8-dihydroguanosine; Cr: creatinine.

Figure 1 shows that urinary 8-oxo-dGsn/Cr slightly changed at each stage, but statistically significant differences were not found. Plasma 8-oxo-dGsn was not detected because of the low level of 8-oxo-dGsn in blood.

**Figure 2:**
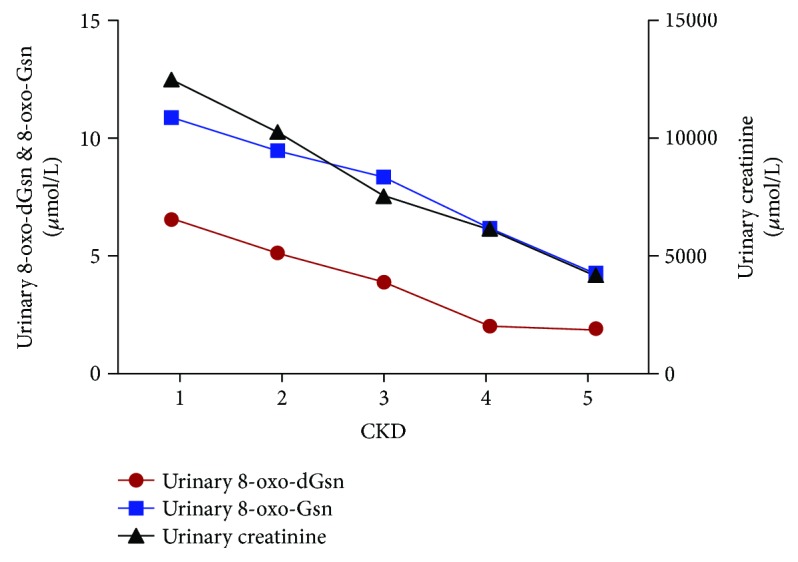
The concentration of urinary 8-oxo-dGsn, 8-oxo-Gsn, and creatinine in CKD patients. CKD: chronic kidney disease; 8-oxo-dGsn: 8-oxo-7,8-dihydro-2′-deoxyguanosine; 8-oxo-Gsn: 8-oxo-7,8-dihydroguanosine; Cr: creatinine.

The changes in concentration of urinary 8-oxo-dGsn, 8-oxo-Gsn, and creatinine in all stages of patients are shown in Figure 2. Due to the variability among the urinary volumes and the significant differences in the renal glomerular function, 8-oxo-dGsn and 8-oxo-Gsn levels were normalized relative to the amount of creatinine. The results show that the two urinary biomarkers had a positive correlation when standardization with creatinine was made. The correlation analysis is shown in Table 3.

### 3.3. The Ratio of 8-oxo-Gsn in Urine to Plasma Was Consistent with eGFR

**Figure 3:**
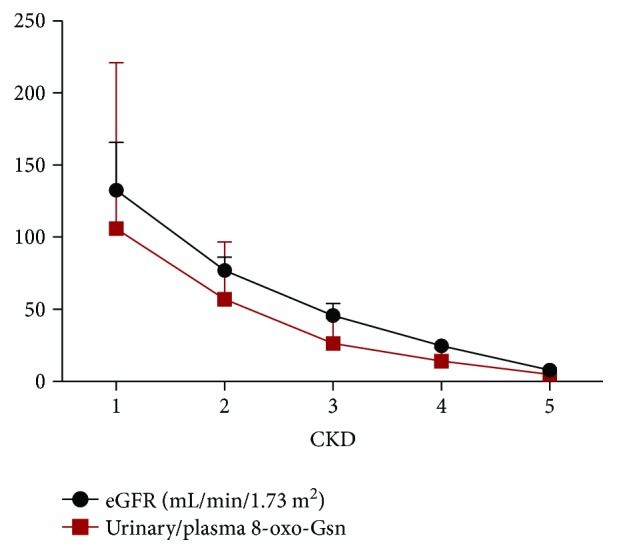
The eGFR and the ratio of 8-oxo-Gsn in urine and plasma in CKD patients. Spearman's correlation analysis was performed and the coefficient (*r*) is 0.779 (*P* < 0.01). CKD: chronic kidney disease; eGFR: estimated glomerular filtration rate; 8-oxo-dGsn: 8-oxo-7,8-dihydro-2′-deoxyguanosine; 8-oxo-Gsn: 8-oxo-7,8-dihydroguanosine; Cr: creatinine.

**Figure 4:**
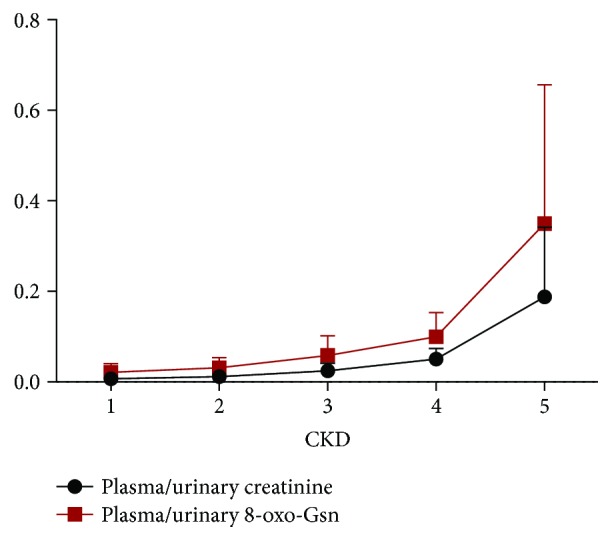
The ratio of creatinine and 8-oxo-Gsn in plasma and urine in CKD patients. Spearman's correlation analysis was performed and the coefficient (*r*) is 0.888 (*P* < 0.01). CKD: chronic kidney disease; 8-oxo-dGsn: 8-oxo-7,8-dihydro-2′-deoxyguanosine; 8-oxo-Gsn: 8-oxo-7,8-dihydroguanosine; Cr: creatinine.

The ratio of 8-oxo-Gsn in urine to plasma was consistent with the eGFR, as shown in Figure 3, and the coefficient (*r*) was 0.779 (*P* < 0.01).  Figure 4 shows that between stage 4 and stage 5, the ratio of 8-oxo-Gsn in plasma to urine increased by 289%, more than that of creatinine, which increased by 260%.

### 3.4. Influence Factor of 8-oxo-dGsn and 8-oxo-Gsn Levels

8-oxo-dGsn and 8-oxo-Gsn levels can be influenced by age. In fact, they have been historically used as indicators of aging. Considering this problem, we analyzed the influence of age and the renal function on plasma 8-oxo-Gsn and the ratio of 8-oxo-Gsn in plasma to urine with multiple regression analyses. Plasma 8-oxo-Gsn = serum creatinine × 0.656 − 0.233 (*P* = 0.000). The ratio of plasma to urine 8-oxo-Gsn = serum creatinine × 0.550 − 0.148 (*P* = 0.000). Results showed that renal function was an independent risk factor.

## 4. Discussion

The largest cause of mortality in CKD patients is cardiovascular disease, especially in patients with ESRD. In the past decade, many efforts have been made to determine the causative or associated factors that contribute to high mortality from cardiovascular disease. Traditional risk factors, such as hypertension and hypercholesterolemia, were unable to account for the high-mortality rate from cardiovascular disease [9, 10].The hypothesis that oxidative stress plays an important role was, therefore, accepted by most scholars. Patients with CKD are particularly vulnerable to oxidative stress. From the early stages of the disease, this population is exposed to a multitude of risk factors that are linked to higher oxidative stress [11]. Biomarkers of oxidative stress can be evaluated, including the degradation of lipids, proteins, and nucleic acids. The relationship between the oxidative damage of nucleic acids and disease has recently attracted increasingly more attention. Many studies have found that the level of RNA oxidation is increased in psychological and oxidative stress-related diseases [12–15], diabetes [16], and cancer [17], implying that other diseases [18, 19] may also be affected. A series of studies that focused on type 2 diabetes suggested that 8-oxo-Gsn was an independent predictor of long-term mortality, and the change in urinary 8-oxo-Gsn was associated with increased mortality [20, 21]. Our study also showed that nucleic acid oxidation could predict the occurrence of diabetic nephropathy and may be a sensitive indicator of early-stage disease.

To our knowledge, the changes in nucleic acid oxidative damage in renal disease have not been investigated. A study of DNA oxidation in dialysis patients has been reported, and patients in the predialysis group showed higher values for most of the oxidized molecules. The peritoneal dialysis group showed a better oxidation-antioxidation balance, with no significant differences in levels of mitochondrial 8-oxo-dGsn when compared to the control group [3]. This suggested that nucleic acid oxidation plays an important role in renal disease. In the current study, we found that 8-oxo-Gsn increased gradually with the deterioration of renal function in patients with CKD. The levels of plasma 8-oxo-Gsn and urinary 8-oxo-Gsn/Cr were positively correlated with the deterioration of renal function in CKD stages 1–4. These results indicate that nucleic acid oxidation plays a very important role in renal function damage or that a decrease in renal function could cause nucleic acid oxidative stress in CKD patients. Antinucleic acid oxidation could be an effective treatment in prolonged renal damage.

However, the unexpected result was that urinary 8-oxo-Gsn/Cr did not increase but, in fact, declined in stage 5 CKD patients. We theorized that this could be related to the lower excretion ability of the kidneys. Severe damage of renal function may affect the excretion of 8-oxo-Gsn, a phenomenon evident in proteinuria. We found that proteinuria decreased in ESRD patients. The glomerular filtration membrane has a three-layer structure, which (from the inside out) consists of the following: endothelial cells, the basement membrane, and epithelial cells of the capsule (podocytes). There are pores between the endothelial cells, which are about ~500 to 1000 Å. Small solutes and water can easily pass through these holes. The basement membrane is a continuous dense structure with no holes and a thickness of ~3200 to 3400 Å. The membrane surface is coated with a negatively charged binding substance, the main component of which is a mucopolysaccharide. Long thin gaps exist between the podocytes, and the gap widths are about ~100 to 400 Å, with lengths of about ~200 to 900 Å. Only molecules equal to or less than the size of albumin (approximately 68,000 Da) are able to pass through the glomerular filtration membrane pores. The molecular weights of the oxidation products are very small. The weight of 8-oxo-dGsn is approximately 283 Da, and 8-oxo-Gsn is approximately 299 Da, and they are freely able to pass through the kidney like creatinine, whose weight is approximately 113 Da. However, no research has reported the exact metabolism pathway until now. The oxidation products 8-oxo-dGsn and 8-oxo-Gsn can be produced by all kinds of cells, tissues, and organs. The results of our previous report suggested that larger amounts of 8-oxo-dGsn and 8-oxo-Gsn are detected in urine [22, 23]. Levels of 8-oxo-dGsn and 8-oxo-Gsn in urine were positively correlated with those in blood, plasma, and serum. All of these phenomena suggest that 8-oxo-dGsn and8-oxo-Gsn can be excreted by the kidneys and can thus reflect the levels of DNA or RNA oxidative stress of the entire body. As for our previous study (paper in press), the 8-oxo-dGsn and 8-oxo-Gsn levels in random urine samples could replace those in 24 h urine samples and were considered as being representative of the level of systemic oxidative stress for the entire day. The plasma levels of 8-oxo-Gsn and the increase in the ratio of plasma to urine 8-oxo-Gsn in stage 5 patients confirm that nucleic acid oxidation was in association with renal damage and renal failure affected excretion of nucleic acid oxidative products.

RNA may have higher levels of oxidative lesions compared with DNA. Reasons for this may include the following: RNA is a single-stranded nucleic acid without the protection of histone proteins, RNA polymerase lacks the correction function, and RNA is widely distributed and highly expressed in cells [24–26]. DNA is the primary material for storage, replication, and transmission of genetic information. RNA is a bridge in the process of genetic information transfer, which is the translation of the genetic information into protein. Thus, RNA oxidation appears to be more relevant. The most suitable marker for studying RNA oxidation is 8-oxo-Gsn. Recently, many researchers have found RNA oxidation to be one of the hallmarks in the pathogenesis of many diseases. In our study, we did not find a significant increase of 8-oxo-dGsn in CKD patients. This result confirmed that RNA oxidation plays a more important role in CKD patients.

The plots of urinary nucleic acid oxidation products and creatinine concentration were consistent, as shown in Figure 2. The eGFR is recognized as an evaluation index for renal filtration function. We found that the ratio of 8-oxo-Gsn in urine and plasma was similar to eGFR, which means that 8-oxo-Gsn could be used as a suitable marker for renal function. Interestingly, the level of 8-oxo-Gsn in plasma and urine was more markedly increased than that of creatinine in stage 5. We know that serum creatinine as a measure of renal function has some limitations. Our results indicated that 8-oxo-Gsn was more sensitive than creatinine for evaluating ESRD.

8-oxo-Gsn was first studied as an index of aging. In our research, patients with stage 1 and stage 2 CKD were younger. This was a clinical characteristic of the disease that we could not control. We analyzed the influence of age with regression analyses and found that 8-oxo-Gsn was only related to renal function.

In conclusion, (1) RNA oxidative damage is present in patients with renal disease and increases with deterioration of the disease. (2) The level of 8-oxo-Gsn in plasma and urine is a novel evaluation index of ESRD.

There are still many limitations in our research, for example, the excretion of 8-oxo-Gsn via the kidneys is still speculation, and more experimental evidence is needed. In addition, the sample size was small, and it was a single-center study in a Chinese population. Therefore, additional studies are required to confirm the results presented here.

## Figures and Tables

**Table 1:** Demographic and clinical characteristics of the study population.

	CKD 1 *n* = 30	CKD 2 *n* = 30	CKD 3 *n* = 31	CKD 4 *n* = 30	CKD 5 *n* = 25
*Demographic characteristics*					
Age (years)	41.63 ± 14.38^∗^	53.13 ± 17.77^∗^	61.47 ± 11.05	63.23 ± 16.38	63.96 ± 12.28
Male sex (%)	13 (43.3)	17 (56.7)	14 (45.2)	18 (60)	8 (32)
*Clinical characteristics*					
PRD	26	25	15	18	18
SRD	4	3	12	10	9
TIN	1	1	3	2	3
Others	0	1	1	0	0

The data are expressed as the mean ± SD; ^∗^ *P* < 0.05. CKD: chronic kidney disease; PRD: primary renal disease; SRD: secondary renal disease; TIN: tubular interstitial nephropathy.

**Table 2:** Creatinine and products derived from nucleic acid oxidation.

	CKD 1	CKD 2	CKD 3	CKD 4	CKD 5
Plasma creatinine (*μ*mol/L)	61.60 ± 14.21^a^	90.33 ± 26.26^ab^	142.87 ± 63.91^b^	202.03 ± 53.21^b^	590.40 ± 277.87^bc^
Urinary creatinine (*μ*mol/L)	12,494 ± 6926.21^a^	10270.07 ± 6238.45^a^	7359.17 ± 3882.42^b^	6447.93 ± 4354.35^bc^	4268.64 ± 5782.24^bc^
eGFR	132.53 ± 33.21^a^	76.98 ± 9.1^b^	45.81 ± 8.5^c^	24.73 ± 4.56^d^	7.84 ± 2.9^e^
Urinary 8-oxo-dGsn (*μ*mol/L)	6.56 ± 5.11^a^	5.14 ± 3.54^a^	3.97 ± 2.58^ab^	2.03 ± 1.46^b^	1.92 ± 2.04^b^
Urinary 8-oxo-Gsn (*μ*mol/L)	10.87 ± 6.15^a^	9.49 ± 4.74^ab^	8.39 ± 5.52^ab^	6.17 ± 3.78^bc^	4.29 ± 2.62^bc^
Urinary 8-oxo-dGsn/Cr (*μ*mol/mol)	1.87 ± 0.87	1.92 ± 1.01	1.84 ± 0.81	1.47 ± 0.89	1.60 ± 1.59
Urinary 8-oxo-Gsn/Cr (*μ*mol/mol)	3.07 ± 1.07^a^	3.42 ± 1.34^a^	3.72 ± 1.47^a^	3.90 ± 1.93^b^	3.75 ± 2.26^a^
Plasma 8-oxo-Gsn (*μ*mol/L)	0.17 ± 0.12^a^	0.24 ± 0.18^ab^	0.37 ± 0.20^b^	0.49 ± 0.22^bc^	1.10 ± 0.57^bcd^
Plasma/urinary 8-oxo-Gsn	0.02 ± 0.02^a^	0.03 ± 0.02^a^	0.06 ± 0.04^a^	0.10 ± 0.05^b^	0.34 ± 0.03^bc^

The data are expressed as the mean ± SD; letters indicate statistical significance (*P* < 0.05). CKD: chronic kidney disease; eGFR: estimated glomerular filtration rate; 8-oxo-dGsn: 8-oxo-7,8-dihydro-2′-deoxyguanosine; 8-oxo-Gsn: 8-oxo-7,8-dihydroguanosine; Cr: creatinine.

**Table 3:** Spearman's correlation analysis between urinary biomarkers of oxidative damage and creatinine.

	8-oxo-dGsn	8-oxo-Gsn
8-oxo-Gsn	0.744^∗∗^	
Creatinine	0.564^∗∗^	0.630^∗∗^

The Spearman's coefficient (*r*) is shown. Urinary biomarkers of nucleic acid oxidation and creatinine are expressed as *μ*mol/L; ^∗∗^ *P* < 0.01. CKD: chronic kidney disease; eGFR: estimated glomerular filtration rate; 8-oxo-dGsn: 8-oxo-7,8-dihydro-2′-deoxyguanosine; 8-oxo-Gsn: 8-oxo-7,8-dihydroguanosine; Cr: creatinine.

## Supplementary Materials

Supplementary Materials Table S1: LC conditions for urine. Table S2: conditions for different compounds in three samples. Table S3: conditions for different samples. Figure S1: the levels of plasma and urinary creatinine in CKD patients. CKD: chronic kidney disease.
