# Supplementary material for: The Ratio of Plasma and Urinary 8-oxo-Gsn Could Be a Novel Evaluation Index for Patients with Chronic Kidney Disease
Source: Oxid Med Cell Longev. 2018 Jan 17;2018:4237812. doi: 10.1155/2018/4237812 (PMC5822777; doi:10.1155/2018/4237812)
Supplement: Supplementary Materials — Table S1: LC conditions for urine. Table S2: conditions for different compounds in three samples. Table S3: conditions for different samples. Figure S1: the levels of plasma and urinary creatinine in CKD patients. CKD: chronic kidney disease. [file 4237812.f1.docx]

**Supplementary Material**

Table S1. LC conditions for urine

| Sample | Temperature | Flow | The percentage of A in relation to time | | | | |
| --- | --- | --- | --- | --- | --- | --- | --- |
|  |  |  | 90%-70% | 70%-2% | 2%-2% | 2%-90% | 90%-90% |
| urine | 35 °C | 0.4 mL/min | 0-3 min | 3-4 min | 4-5 min | 5-5.01min | 5.01-7min |

Table S2. Conditions for different compounds in three samples

| sample | Compounds | Mass transitions | Internal standard mass transitions | Collision energy | Fragmentor voltage | Cell accelerator voltage |
| --- | --- | --- | --- | --- | --- | --- |
| urine | 8-oxo-dGsn | m/z 284-168 | m/z 289-173 | 10 eV | 380 V | 3 |
|  | 8-oxo-Gsn | m/z 300-168 | m/z 303-171 | 14 eV | 380 V | 3 |

Table S3. Conditions for different samples

| Type of sample | Gas temperature | Gas flow | Sheath gas temperature | Sheath gas flow | Nebulizer | Capillary | Nozzle voltage | High pressure RF | Low pressure RF |
| --- | --- | --- | --- | --- | --- | --- | --- | --- | --- |
| urine | 200 °C | 16 L/min | 400 °C | 12 L/min | 30 psi | 2000 V | 0 | 120 V | 50 V |

Fig S1. The levels of plasma and urinary creatinine in CKD patients. Abbreviation CKD: chronic kidney disease.

Fig S1
